# Fear of Missing Out, Emotional Intelligence and Attachment in Older Adults in Argentina

**DOI:** 10.3390/jintelligence11020022

**Published:** 2023-01-21

**Authors:** Marian Durao, Edgardo Etchezahar, Miguel Ángel Albalá Genol, Mariela Muller

**Affiliations:** 1 Faculty of Education, International University of Valencia, 46002 Valencia, Spain; 2 Faculty of Psychology, Universidad de Buenos Aires, Buenos Aires 1053, Argentina; 3 National Scientific and Technical Research Council (CONICET), Buenos Aires 1428, Argentina

**Keywords:** fear of missing out, emotional intelligence, FoMO, older adults, attachment, emotional intelligence, depression, interpersonal relationships

## Abstract

In recent years, the rise of social networks has changed relationships and lifestyles around the world. This has led to the emergence of the Fear of Missing Out (FoMO), which consists of the need to constantly check social media and the anguish that comes from feeling a lack of rewarding experiences. The impact derived from the use of technologies in a digital environment has been widely studied in young people but not so much in older adults. The main aim of this study was to analyze FoMO levels in older adults and their relationships with sociodemographic and formative factors. Another aim of this study was to analyze whether the dimensions of emotional intelligence, the different forms of attachment and psychological symptomatology affect the FoMO levels of older adults. A total of 690 older adults from Argentina aged between 60 and 90 years (M = 69.01; SD = 5.48) participated, 54.5% of whom were women, responding using a geolocated online questionnaire. The main results confirmed that older people show FoMO levels similar to other general samples. In addition, results show several predictor variables with respect to FoMO: emotional attention, insecure attachment, depression and difficulties in interpersonal relationships. The implications of the results observed in older adults are discussed.

## 1. Introduction

In recent years, the exponential growth in the use of technology has significantly facilitated the reduction of social and emotional distance between people, particularly since the emergence of COVID-19. In this situation, older adults (people over 65 years of age) were affected (Haase et al. 2021) because of the risk of suffering mental health problems derived from social isolation (Oddone and Pochintesta 2021; OMS 2021).

Despite the obvious benefits of social media and the digital world, they are not without their challenges in the area of mental health (Scheinfeld and Voorhees 2022). One of the negative aspects of the use of social media is the so-called Fear of Missing Out (FoMO): the desire to be permanently informed of what is happening to others on social networking sites, which leads to an excessive use of social networks, associated with the belief that others are experiencing more pleasurable situations than one’s own (Przybylski et al. 2013). FoMO leads to various cognitive and emotional aspects that affect mental health and social relationships (Hayran and Anik 2021). Recent studies in the Argentine population (Cohen et al. 2020; Cervigni et al. 2022) reported a significant increase in symptoms related to various psychological disorders (e.g., a 43% increase in anxiety, a 29% increase in depression, among others) and social isolation in all age groups.

So far, the study of FoMO has been conducted mainly in a young population. However, older adults have significantly increased their use of technologies and their various functionalities (Fernández and Guglialmelli 2021; Liu et al. 2016; Zhang et al. 2021), necessitating their study, specifically in the older population.

### 1.1. Emotional Intelligence as a Possible Protective Factor of FoMO

Emotional intelligence (hereafter EQ) comprises a set of intrapersonal and interpersonal competencies and skills, which influence the way in which individuals relate to their community (Ruvalcaba et al. 2014). According to Goleman (1995, p. 68), it constitutes “a meta-skill that determines the degree of ability to master our other faculties” (Goleman 1995, p. 68). According to this model, emotional intelligence includes (Mayorga-Lascano 2019): attention, i.e., the degree to which people consider that they are aware of their own emotions and feelings; emotional clarity, the way in which people believe they perceive their own emotions; and emotion repair, i.e., the belief in one’s own possibility to regulate one’s emotional states.

There is evidence that EQ is a predictor of both physical and mental health (Schutte et al. 2007; Petrides et al. 2016). In this sense, it is a protective factor against addictions or violent behaviors (González Yubero et al. 2021; Orozco Solis 2021), and it is also related to well-being in older adults, favoring critical thinking, promoting inclusion and modulating FoMO (Bandera-Pastor et al. 2022). Likewise, the relationship between cell phone use and EQ, or between FoMO and EQ, has been documented in numerous studies (Mosa Qutishat 2020). In recent years, various studies have shown the relationship between emotional intelligence and FoMO, which is often considered a protective factor. However, these studies have been conducted mainly in the adolescent and university population or in the general population (Soriano-Sánchez 2022). 

Regarding the link between emotional intelligence and FoMO, in young people, it has been shown that it favors academic success and a beneficial use of social media (Iqbal et al. 2021). Emotional intelligence, besides being negatively related to FoMO, acts as a mediator with respect to depression (Kartol and Gündoğan 2020). Thus, there are numerous studies (Casale et al. 2018 ; Hamissi et al. 2013; Van Deursen et al. 2015) that have found negative relationships between EQ and FoMO in young and adult populations, as well as with respect to other variables associated with the development of FoMO (internet and cell phone addiction and abuse, social anxiety, self-regulation, among others). From an applied perspective, Chen et al. (2022) explored the effect of psychological interventions on FoMO that improve emotional intelligence, finding improvements and a reduction of FoMO. 

Given the cross-cutting impact of FoMO and emotional intelligence on the entire population, it is necessary to develop studies that specifically consider the population of older adults, thus evaluating the possible differences with respect to the populations already studied. Thus, to guarantee an adequate study of this variable, it is necessary to consider a population with a heterogeneous geographic distribution and a population which is not only represented by large cities (Cole 2009). Therefore, heterogeneity and greater representativeness in relation to the study are favored, taking into account the population from various locations in the country (Argentina).

### 1.2. Forms of Attachment and Their Implications with Regards to FoMO

In recent years, assorted studies (Blackwell et al. 2017) have shown evidence of the role of distinct attachment profiles in the development of FoMO and other related behaviors. According to attachment theory (Ainsworth 1963; Bowlby 1969), the bond established between a child at an early age and its caregivers lays the foundation for the emotional attachment style of the adult individual. The effects of childhood attachment relationships extend into adulthood, and influence the emotions, relational style and defensive behaviors of individuals (Bowlby 1984) throughout their lives (Soriano-Sánchez 2022). The aforementioned leads to the classification of attachment most commonly used in the psychological field (Bowlby 1984; Main and Solomon 1986) into: secure, avoidant, anxious and ambivalent. 

First, secure attachment is shown in adulthood through the ability to have healthy relationships with other people, where an environment of intimacy and closeness can be developed, with adequate autonomy, self-efficacy and self-esteem. Secondly, avoidant attachment is shown as a rejection towards commitment, in which the person may feel a certain fear or refusal to manage relationships that have a degree of intimacy, generating discomfort in them and preferring independence. Thirdly, in the anxious attachment style, the person has a constant need for contact in their relationships, feeling a great discomfort derived from the lack of reciprocity. Finally, ambivalent attachment appears as a great fear of abandonment or loneliness, which generates situations of dependence due to the low personal autonomy developed and the need for constant approval. 

Apparently, subjects with an insecure attachment style (anxious, avoidant or ambivalent) would have behaviors that tend to replace the affection of those close to them (i.e., family and friends) through the search for gratification in social media (D’Arienzo et al. 2019; Holte and Ferraro 2020). Specifically, in relation to FoMO and its link with attachment styles, both are significantly related. According to previous studies, FoMO is found to be related to the anxious attachment style rather than the avoidant attachment style. Meanwhile, Blackwell et al. (2017) noted among his findings that anxious and ambivalent attachment styles predict the abusive use of modern technologies; in other words, people often seek to soothe their discomfort through the use of social media. Furthermore, those who show avoidant attachment, although they consider themselves self-sufficient and frequently avoid intimacy, also want to use social media as a way to keep people in their lives but at a distance (Aranda et al. 2022; Nitzburg and Farber 2013). Likewise, people with the ambivalent attachment style are at a greater risk of developing addictive behaviors (Valizadeh et al. 2017). High FoMO levels lead to detachment in adult relationships (Hogan 2015). Therefore, it is necessary to continue to study in depth the degree and type of relationship between the different attachment styles and the development of FoMO and other related variables.

### 1.3. Psychological Symptoms in FoMO

In recent years, older adults increased their use of technology and social media, sometimes promoted by family and community support (Galica et al. 2021) to mitigate the consequences of social disconnection and its consequences on mental health deterioration, especially during the COVID-19 pandemic (Merchant and Lurie 2020). Many older adults knew how to use technology to connect with others prior to the onset of the pandemic, and many others adopted new technologies from the onset of the pandemic and pointed out problems related to dependence on family and friends due to lack of knowledge (Haase et al. 2021).

However, there is compelling evidence that social connectedness facilitated by the use of technology improves the mental, social and physical well-being of older adults (Baker et al. 2018; Siegel and Dorner 2017). Still, at present, many studies report new problems generated by the excessive use of these technologies (Akbari et al. 2021). Some groups may be more oriented to develop FoMO, such as those characterized by excessive use of social media, regardless of their stage in life. Thus, FoMO may increase with age in some populations (Akbari et al. 2021).

The mechanism underlying FoMO is shaped by cognitive and anxiety-related aspects (Przybylski et al. 2013), which are reflected in subjective discomfort, interpersonal difficulties, depression, problematic use of social media, attention and concentration difficulties, and these aspects also hinder the life experience (Przybylski et al. 2013; Hayran and Anik 2021). The desire to be permanently informed of what is happening to others on social media can generate problems in family interactions (Scheinfeld and Voorhees 2022) and higher levels of loneliness and envy in adults (Barry and Wong 2020), generating social comparisons based on the information they see on social media, which triggers the belief that others are doing something fun and are happier, with a consequent decrease in self-esteem (Buglass et al. 2017).

Specifically, the appearance and development of FoMO is related to a discomfort frequently linked to psychological symptoms (depression, anxiety, loneliness, self-esteem problems, among others) that affect people’s well-being; the appearance and development of FoMO is also related to the abusive use of social media and smartphones (Sánchez-Carbonell et al. 2008). Several studies have empirically corroborated the predictive effect of anxious-depressive, psychotic and interpersonal sensitivity symptoms on FoMO and problematic smartphone use (Gul et al. 2022; Tugtekin et al. 2020), although the sample of participants is usually limited to young people. Thus, the need to investigate in older adults the relationship between FoMO and psychological symptomatology, as well as its effects, is evident.

In accordance with the above, this work had two main objectives: First, to analyze FoMO levels in a sample of older adults and their relationships with gender, age, social class and educational level. Second, to study whether the dimensions of emotional intelligence, different forms of attachment and psychological symptoms affect the levels of FoMO in older adults. Based on the objectives of the study, two hypotheses were raised. On the one hand, according to hypothesis 1, it is expected that FoMO will be found to be present in older people with levels similar to those of other groups of the general population. In this way, aging is not a process that gives rise to these new problems, but rather there will be other psychosocial and socio-emotional variables that need to be analysed. Therefore, it is necessary to study these novel variables in the older population as well as in samples of young people. On the other hand, according to hypothesis 2, the variables studied will show relationships in different directions in relation to FoMO; emotional intelligence will be related in a negative sense, as well as the type of secure attachment. In contrast, it is expected to find positive relationships between FoMO and insecure attachment styles, as well as with the psychological symptoms explored. These relationships with psychosocial variables will therefore be relevant to explain the levels of FOMO in older people.

## 2. Materials and Methods

### 2.1. Participants

A geolocalized online questionnaire was administered, with stratified sampling based on the geographical regions of Argentina (see Table 1). The complete and valid protocols totaled 690 cases (with a sampling error of ±2.5% and a confidence level of 95%) from urban environments. The average age of the participants was 69.01 years (SD = 5.48), with an age range between 60 and 80 years. We performed a post hoc power analysis (Power = Φ (−z1 − α/2 + |μ0 − μ1| ^x^n√n/σ), taking into account about two million people between 60 and 80 who reside in Argentina according to the INDEC (2018), and the sample size was adequate (Φ (26,265.858) = 1 = 100% power. Of the participants, 54.5% (n = 376) identified as female, while 45.5% (n = 314) identified as male. With respect to educational level, 4.6% of the sample had only primary education, 26.2% had secondary education, 31.2% had tertiary education and 38% had completed their university studies.

### 2.2. Measures

Self-report measures were employed using a battery of assessment instruments consisting of:

*Fear of Missing Out (FoMO) Scale.* To assess the construct, we proceeded to perform the adaptation and validation of the original version of the scale (Przybylski et al. 2013), composed of 10 items that determine dimension 1, FOM-NI (e.g., “I fear my friends have more rewarding experiences than me”) and dimension 2, FOM-SO (e.g., “It bothers me when I miss an opportunity to meet up with friends”). Each item was rated on a Likert scale with five anchors, ranging from 1 = Strongly disagree to 5 = Strongly agree (the same response format was used for the other scales used in this study). The higher the score in both dimensions, the higher the levels of FoMO.

*Trait Meta-Mood Scale (TMMS-12).* A reduced 12-item version of the 12-item Trait Meta-Mood Scale proposed by Salguero et al. (2009) was used, which was derived from the TMMS-24 (Salovey et al. 1995; Fernández-Berrocal et al. 1998). This scale consists of 12 items, which determine three dimensions. The first dimension, emotional attention, represents the ability to monitor one’s own emotions and feelings (e.g., “I pay a lot of attention to the way I feel”); the second dimension, clarity of emotions, refers to the ability to understand and label one’s emotions (e.g., “I can usually define my feelings”); and finally, the third dimension, emotional repair, represents the ability to regulate negative emotional states and prolong the positive ones (e.g., “Although I sometimes feel sad, I usually have an optimistic outlook”). A Likert-type response format ranging from 1 = Strongly disagree to 5 = Strongly agree was used in this study.

*Relationship Questionnaire (RQ).* A four-item scale designed to measure attachment style in adults was used. This questionnaire is an adapted version of the original, which extends the three measures of attachment (Shaver and Hazan 1987) to four. The version adapted to Spanish, proposed by Alonso-Arbiol et al. (2002) consists of a total of four items, each of which represents an attachment style: secure attachment (“It is easy for me to become emotionally close to others. I am comfortable depending on them and having them depend on me. I don’t worry about being alone or having others not accept me.”); avoidant attachment (“I am comfortable without close emotional relationships. It is very important to me to feel independent and self-sufficient, and I prefer not to depend on others or have others depend on me.”); anxious attachment (“I want to be completely emotionally intimate with others, but I often find that others are reluctant to get as close as I would like. I am uncomfortable being without close relationships, but I sometimes worry that others don’t value me as much as I value them.”); and ambivalent attachment (“ I am uncomfortable getting close to others. I want emotionally close relationships, but I find it difficult to trust others completely, or to depend on them. I worry that I will be hurt if I allow myself to become too close to others.”). Each item is rated on a Likert scale with five anchors, ranging from 1 = Strongly disagree to 5 = Strongly agree.

*Online Global Symptoms Scale (Escala de Síntomas Globales Online or ESGO):* The local version of the ESGO was used to evaluate the construct (Durao et al. 2019), composed in its definitive version of 20 items that account for four dimensions that make up the global evaluation of the symptomatology presented by the individuals. For our study, three of the four dimensions were used; psychoticism was discarded, since it does not have a theoretical link with the study variables. Each of the dimensions is made up of a series of five items: depression (e.g., “I often feel that my problems are unsolvable”; “I don’t enjoy myself as much as I used to”), anxiety (e.g., “At times I feel intense fear and think I’m going to die”; “I have sensations in my body that others can’t understand”), interpersonal relations (e.g., “I tend to be on the lookout for negative judgment”; “I sometimes feel inferior to my peers or friends”). Each item is rated on a Likert scale with five anchors, ranging from 1 = Strongly disagree to 5 = Strongly agree.

*Socio-demographic data questionnaire:* Information on the gender, age, self-perceived socio-economic level, household typology (lives alone/does not live alone) and highest level of education was collected from the participants.

### 2.3. Procedure and Data Analysis

People who met the criteria of age (60 and over) and geographic region were invited to participate via social media, based on the quotas stipulated for the sample distribution. Participants were previously informed, at the start, about the purpose of the study, the institution responsible for it and they were provided with a contact e-mail address in case they required further information. Additionally, they were informed that the data collected in this study would only be used for academic-scientific purposes and would be protected in accordance with Argentine National Law 25,326 on the protection of personal data. The questionnaires were collected from March to May 2022. The statistical analyses that guided the development of this study were conducted using SPSS for Windows software version 19.0 (George and Mallery 2010) and EQS 6.1 (Bentler 2007). First, a descriptive analysis of the FoMO-NI and FoMO-SO (means and standard deviations) was conducted. Also, Pearson’s correlations have been estimated to analyze the relationship between the FoMO dimensions, educational level and participants’ age. Moreover, an analysis on the relationships between FoMO, emotional intelligence, attachment and psychological symptoms was conducted. Finally, we performed a multiple linear regression on emotional intelligence, attachment types and symptomatology to explain the variance in FoMO-NI and FoMO-SO.

## 3. Results

Firstly, when comparing the results of the general population (Durao et al. 2023) with the sample of older adults, no differences were observed in the FoMO means. Regarding FoMO-NI, in the general population, a mean of 2.21 (DT = 1.053) was observed and FoMO-SO had a mean of 3.14 (DT = 1.131). In our sample of older adults with whom we worked in this study, it was observed that for FoMO-NI, the mean was 2.12 (DT = 0.959) and for FoMO-SO the mean was 3.18 (DT = 1.112).

Next, the relationships between FoMO dimensions and participants’ age, educational level and social class were analyzed (Table 2).

As seen in Table 2, the two dimensions of FoMO are significantly related to each other, but no relationships are observed with other variables such as participants’ age, educational level and social class. Likewise, no differences were observed based on participants’ gender.

Subsequently, we proceeded to analyze the relationships between the dimensions of FoMO and emotional intelligence, the various expressions of attachment and psychological symptoms (Table 3).

Finally, a stepwise regression model was tested to analyze which variables make the greatest contribution to FoMO in older adults (Table 3). To this end, different models were calculated, some considering FoMO-NI as the dependent variable and others considering FoMO-SO as the dependent variable. As independent variables, we considered, first, the three dimensions of emotional intelligence, second, the different forms of attachment and, finally, three categories of psychological symptoms.

As seen in Table 4, for FoMO-NI and FoMO-SO, the first dimension, emotional intelligence, is fundamental, since it makes a significant contribution to both dimensions; however, the other two dimensions do not make any contribution to emotional intelligence. Regarding the different forms of attachment, for FoMO-NI, attachment 3 (AT-Anx) and 4 (AT-Amb) are significant, while for FoMO-SO, the contribution of attachment 1 (AT-Secure) is significant. Finally, symptomatology is relevant for FoMO-NI, particularly depression (Deppres.) and interpersonal relationships (Interp. Rel.), which is not the case for FoMO-SO since the change in R-squared is not significant for step 3 and the contribution of interpersonal relationships is very low.

## 4. Discussion

The main objective of this study was to analyze the level of FoMO in older adults and its relationship with various sociodemographic, educational and psychosocial variables. According to the current literature, FoMO has been studied mainly in young people, with older people being an age group that is assumed to be unaffected by this problem, since they are digital migrants (Román-García et al. 2016). However, one of the main findings of the present study showed that there are no significant differences in FoMO levels between older adults and the general population. This seems to indicate that FoMO is a phenomenon that affects all age segments and, therefore, it is necessary to study its possible consequences on the older population. In this sense, and despite the fact that current empirical studies are mostly focused on adolescents and young adults, several authors suggest that FoMO levels increase with age; it is, thus, present in all age groups (Casali and Torres 2021; Fernández and Guglialmelli 2021; Liu et al. 2016; Zhang et al. 2021).

The two dimensions of the FoMO construct in our study replicate the findings of Li et al. (2022): on one hand, the dimension called “Fear of missing novel information” (FOM-NI), and on the other, the dimension called “Fear of missing social opportunities” (FOM-SO). In our sample of older adults, we observed that the values for both dimensions were similar to those found in previous research with adults and young people in similar contexts (Durao et al. 2023). This fact could be derived from the increase in the daily use of technologies (with different functions not only linked to leisure) that has occurred across the population, regardless of their age (Haase et al. 2021), but it may suppose a different scope for the social functioning of the older population. While technology is considered, by younger people, as just another option that enriches social relationships, occasionally for the older population, it could be rather an imposition as the only way of interaction. Based on the first hypothesis, these results are consistent with those evidenced in the correlational analysis, since age did not show relationships with either of the two FoMO factors. On the one hand, FOM-NI seems to be related to the fear that other people are having more rewarding experiences because of the constant updating of information. In this sense, the COVID-19 pandemic may have caused the population of older adults to rely on the people in their environment (Banskota et al. 2020), needing help to connect and participate in virtual meetings and dealing with technical difficulties. On the other hand, FOM-SO is linked to difficulties related to the comprehension of the communicative codes of interaction with respect to the groups to which people belong in their lives. FoMO and the lack of understanding and interpretation of social media may become sources of stress and anxiety (Gil et al. 2015), and even an indicator of psychological and social distress (Vivaldi and Barra 2012).

Based on the second hypothesis, the relationships between FoMO and the analyzed constructs were explored and contrasted. With respect to emotional intelligence (EQ), the findings point to a relationship of FOM-NI with the attention dimension of EQ. In this regard, attention would be involved in the process of constantly checking various digital and technological media in order to have a greater sense of being up-to-date. Furthermore, based on the results, in all cases, attention is a predictor variable of FOM-NI and FOM-SO. Therefore, the ability to monitor one’s own emotions and feelings acts as a predictor of various FoMO-related behaviors in older adults. Attention may be a protective factor against negative symptoms and addictive tendencies or impulsive behaviors (González Yubero et al. 2021; Orozco Solis 2021) in addition to being a protective factor for FoMO and enhancing critical thinking (Bandera-Pastor et al. 2022; Sporzon and López-López 2021). However, high emotional attention values by themselves may generate hypervigilance if the other factors of emotional intelligence (emotional clarity and repair) are not developed. Additionally, we only found a predictive effect of FoMO with respect to the clarity of emotions dimension (EQ) in one of the contrasted models. It is necessary to consider the great relevance of contact and social interactions for the correct development of emotional intelligence (Ruvalcaba et al. 2014), which could be weakening the protective value against FoMO in the older population, as evidenced in studies with younger populations. Considering that improving EQ globally decreases FoMO (Chen et al. 2022), there remains a need to evaluate new intervention models to empirically assess whether improvement in the three dimensions (attention, clarity and repair) could function as a protector of FoMO. 

Regarding the relationships between attachment styles and FoMO, the FOM-NI dimension is related to anxious and ambivalent attachment styles. These results are consistent with previous studies (Liu and Ma 2019). In this regard, this bonding is related to the need of people with such attachment styles to be in continuous social contact, combined with avoidant behaviors in the ambivalent type (Aranda et al. 2022; D’Arienzo et al. 2019). In this sense, social media provides a way of social interaction with a certain distance where, additionally, suppressing any type of virtual interaction may be simpler than in face-to-face contexts. In turn, the secure attachment style is related to FOM-SO. This finding could be pointing to possible indirectly positive aspects of FoMO related to the search for social contact and support, although it may manifest with behaviors and consequences that are harmful to the person’s well-being. In the older population, as has been suggested by various organizations and institutions, the use of social media has positive aspects such as facilitating or generating encounters and continuity in interpersonal relationships (Baker et al. 2018; Siegel and Dorner 2017). Thus, the secure attachment type is positioned as a predictor of FOM-SO and not of FOM-NI. However, anxious and ambivalent attachment types appear to be predictors of FOM-NI, which is consistent with several abusive and obsessive behaviors in the use of technologies with the goal of feeling that new information and possible situations are not out of control (Torres Mendoza and Serna Sarrato 2021). Therefore, the secure attachment style affects the presence of FOM-SO more, which can be related to the need to relate to people they are close to and their own social environment. In contrast, insecure attachment styles (anxious and ambivalent) predict FOM-NI, which does not have such a social component and is more related to the need and fear of missing out on novel and rewarding experiences that anyone may be experiencing. In conclusion, in our sample of older people, there is no single attachment style that affects the two dimensions of FoMO in a homogeneous way.

On the other hand, it has been shown that in older adults, FoMO is related to symptoms of depression, anxiety and difficulties in interpersonal relationships. In the case of FOM-NI, depression and difficulties in interpersonal relationships are two clear predictive symptoms. Additionally, only symptoms derived from interpersonal relationships have a predictive value for FOM-SO. In the context of the COVID-19 pandemic, the older population has been affected by illness and has been prone to fear of contracting the virus (Girdhar et al. 2020). This has significantly impacted various indicators of their quality of life and interpersonal relationships (Hall 2021; Cervigni et al. 2022), as well as overall mental health (Mukhtar 2020). While the use of social media has facilitated contact with family and friends for this age group, it can also be inferred that the “Fear of Missing Out” is not only present in the face of what others may be experiencing but also associated with how other older adults do or do not manage social media and technology in general. The use of technology enabled and increased social communication in the older population, reducing feelings of isolation and loneliness (Banskota et al. 2020). This is consistent with what has been stated by several authors; although many individuals approach and remain in social media seeking to mitigate their social exclusion, the same social networks may generate discomfort, loneliness and low self-esteem (Merchant and Lurie 2020), as well as harmful effects and emotions linked to FoMO. Given the deficiencies in social interactions that older people have (Akbari et al. 2021), this could make it the psychological symptomatology with the greatest predictive power on FoMO. However, it is suggested to continue exploring depression and anxiety variables in future studies, as well as other possible ones with a greater presence during aging. 

As has been shown in this paper, it is necessary to continue developing studies to evaluate both FoMO and the presence and impact of other related variables in older adults. Future studies could also delve deeper into differences with respect to gender, age, and even specifically with participants from different occupations. Additionally, it is suggested that in future studies, different variables that could affect people throughout life may be considered, such as unwanted loneliness, feelings of social exclusion, illnesses, among others. Therefore, it would be advisable to know the impact derived from the use of technologies in a digital environment, such as FoMO levels, on the risk of cognitive impairment. It should be noted that no differences were found in terms of household typology (lives alone/does not live alone) with respect to the rest of the variables studied. This aspect should be taken into account in the future. If we intend to improve the levels of psychological and social well-being in the future, it is essential to continue developing studies and strategies that allow us to evaluate all the new changes and phenomena, as well as the psychological impact they have on society, in order to be able to reverse them.

## Figures and Tables

**Table 1:** Distribution of Cases per Region of Argentina.

Geographical Region	f	%	Sex		Age
			Male	Female	
City of Buenos Aires	119	17.3	40.3%	59.7%	60–80 (M = 70.34; SD = 6.35)
Buenos Aires province	337	48.9	46%	54%	60–80 (M = 68.73; SD = 5.22)
Region 1 (Cordoba, Santa Fe, Mendoza, San Luis, San Juan)	110	16	46.4%	53.6%	60–80 (M = 69.59; SD = 5.29)
Region 2 (Salta, Jujuy, Catamarca, La Rioja, Santiago del Estero, Tucuman)	27	3.9	51.9%	48.1%	60–77 (M = 68.11; SD = 4.63)
Region 3 (Misiones, Chaco, Formosa, Entre Rios, Corrientes)	45	6.5	44.4%	55.6%	60–79 (M = 67.40; SD = 5.39)
Patagonian Region (La Pampa, Rio Negro, Neuquen, Chubut, Santa Cruz, Tierra del Fuego)	52	7.4	51%	49%	60–80 (M = 68.65; SD = 5.21)
TOTAL	690	100	45.5%	54.5%	60–80 (M = 69.01; SD = 5.48)

**Table 2:** Relationships between FoMO dimensions, age, educational level and social class.

	1	2	3	4	5
1. FoMO-NI	.802	.312 **	.001	−.091 *	−.063
2. FoMO-SO		.769	−.039	.083 *	.021
3. Age			-	−.018	.148 **
4. Educational level				-	.258 **
5. Social class					-

Note: **. *p* < .001; *. *p* < .01.

**Table 3:** Relationships between FoMO, emotional intelligence, attachment and psychological symptoms.

	M	SD	α	ω	1	2	3	4	5	6	7	8	9	10	11	12
1. FoMO-NI	2.36	0.41	.76	.78	-	.312 **	.354 **	−.094 *	−.126 *	−.016	.006	.364 **	.419 **	.441 **	.401 **	.515 **
2. FoMO-SO	2.41	0.53	.78	.79		-	.314 **	.084	.020	.196 **	−.041	.142 **	.101 *	.056	.115 *	.132 **
3. EI-Attent.	2.77	0.72	.81	.83			-	.187 **	−.024	.063	.054	.289 **	.286 **	.400 **	.436 **	.361 **
4. EI-Clarity	2.65	0.69	.77	.77				-	.345 **	.101 *	.102 *	−.088	−.187 *	−.108 *	−.030	−.170 *
5. EI-Repair	2.91	0.55	.79	.80					-	.091 *	.124 **	−.127 *	−.162 *	−.434 *	−.242 *	−.219 *
6. AT. Secure	2.44	0.61	-	-						-	.140 **	−.030	−.055	.000	−.068	−.052
7. AT-Avoid	2.47	0.54	-	-							-	−.029	.115 **	.061	−.018	−.045
8. AT-Anx.	2.70	0.79	-	-								-	.562 **	.379 **	.326 **	.390 **
9. AT-Amb.	2.81	0.90	-	-									-	.413 **	.355 **	.473 **
10. Deppres.	2.54	0.53	.78	.80										-	.607 **	.536 **
11. Anxiety	2.47	0.73	.81	.82											-	.577 **
12. Interp. Rel.	2.63	0.66	.84	.85												-

Note. α: Cronbach’s Alpha; ω: McDonald’s omega. ** *p* < .001; * *p* < .01.

**Table 4:** Regression model results for the FoMO dimensions.

	Step	Predictor	*β*	*R* ^2^	Δ*R*^2^
FoMO-NI	1	EI-Attent.	0.342 ***	0.133	0.133
		EI-Clarity	−0.120		
		EI-Repair	−0.077		
	2	EI-Attent.	0.209 ***	0.239	0.106 ***
		EI-Clarity	−0.036		
		EI-Repair	−0.065		
		AT- Secure	0.012		
		AT-Avoid.	−0.021		
		AT-Anx.	0.153 **		
		AT-Amb.	0.250 ***		
	3	EI-Attent.	.093 *	0.330	0.91 ***
		EI-Clarity	−.003		
		EI-Repair	.024		
		AT- Secure	.013		
		AT-Avoid.	−.005		
		AT-Anx.	.110 *		
		AT-Amb.	.125 *		
		Deppres.	.139 *		
		Anxiety	.019		
		Interp. Rel.	.291 *		
FoMO-SO	1	EI-Attent.	0.302 ***	0.095	0.095 ***
		EI-Clarity	0.023		
		EI-Repair	0.037		
	2	EI-Attent.	.269 *	0.135	0.039 **
		EI-Clarity	.028		
		EI-Repair	.038		
		AT-Secure	.188 *		
		AT-Avoid.	−.086		
		AT-Anx.	.066		
		AT-Amb.	.012		
	3	EI-Attent.	.283 *	0.145	0.010
		EI-Clarity	.039		
		EI-Repair	−.006		
		AT- Secure	.196 *		
		AT-Avoid.	−.075		
		AT-Anx.	.076		
		AT-Amb.	.018		
		Deppres.	−.128		
		Anxiety	−.011		
		Interp. Rel.	.075 *		

Note: * *p* < .05; ** *p* < .01; *** *p* < .001.

## Data Availability

Data are available from the corresponding author upon reasonable request.
